# Phenotypic and Genotypic Characteristics of *SCN1A* Associated Seizure Diseases

**DOI:** 10.3389/fnmol.2022.821012

**Published:** 2022-04-28

**Authors:** Chunhong Chen, Fang Fang, Xu Wang, Junlan Lv, Xiaohui Wang, Hong Jin

**Affiliations:** Department of Neurology, National Center for Children’s Health, Beijing Children’s Hospital, Capital Medical University, Beijing, China

**Keywords:** epilepsy, sodium channel, *SCN1A*, genetic, generalized epilepsy with febrile seizures plus, severe myoclonic epilepsy of infancy, dravet syndrome

## Abstract

Although *SCN1A* variants result in a wide range of phenotypes, genotype-phenotype associations are not well established. We aimed to explore the phenotypic characteristics of *SCN1A* associated seizure diseases and establish genotype-phenotype correlations. We retrospectively analyzed clinical data and results of genetic testing in 41 patients carrying *SCN1A* variants. Patients were divided into two groups based on their clinical manifestations: the Dravet Syndrome (DS) and non-DS groups. In the DS group, the age of seizure onset was significantly earlier and ranged from 3 to 11 months, with a median age of 6 months, than in the non-DS group, where it ranged from 7 months to 2 years, with a median age of 10 and a half months. In DS group, onset of seizures in 11 patients was febrile, in seven was afebrile, in two was febrile/afebrile and one patient developed fever post seizure. In the non-DS group, onset in all patients was febrile. While in the DS group, three patients had unilateral clonic seizures at onset, and the rest had generalized or secondary generalized seizures at onset, while in the non-DS group, all patients had generalized or secondary generalized seizures without unilateral clonic seizures. The duration of seizure in the DS group was significantly longer and ranged from 2 to 70 min (median, 20 min), than in the non-DS group where it ranged from 1 to 30 min (median, 5 min). Thirty-one patients harbored *de novo* variants, and nine patients had inherited variants. Localization of missense variants in the voltage sensor region (S4) or pore-forming region (S5–S6) was seen in seven of the 11 patients in the DS group and seven of the 17 patients in the non-DS group. The phenotypes of *SCN1A*-related seizure disease were diverse and spread over a continuous spectrum from mild to severe. The phenotypes demonstrate commonalities and individualistic differences and are not solely determined by variant location or type, but also due to functional changes, genetic modifiers as well as other known and unknown factors.

## Introduction

Since the first discovery of *SCN1A* gene associated with epilepsy in 2000 (Escayg et al., 2000), *SCN1A* has remained the most common and important epilepsy pathogenic gene. The gene is located on chromosome 2q^24.3^ and encodes the alpha 1 subunit of the voltage-gated sodium channel Nav1.1, a 2,000 amino acid protein and exhibits dominant interneuron-specific expression. *SCN1A* variants lead to dysfunction of the sodium channel which initiates and propagates neuronal action potentials, thereby causing epilepsy (Stafstrom, 2009). It has an autosomal dominant mode of inheritance with incomplete penetrance. Its pathogenic variants cause a wide range of phenotypes, ranging from the mildest simple febrile seizures (FS) to severe myoclonic epilepsy in infancy (SMEI), or early onset developmental and epileptic encephalopathies (Scheffer and Nabbout, 2019). Although the various phenotypes have distinct characteristics, they often have similar presentations at onset, such as early stage febrile convulsions, thereby making it difficult to predict phenotype development. To date, 2,127 pathogenic variants of the *SCN1A* have been reported and documented in the Human Gene Mutation Database. However, genotype-phenotype correlation of these seizure disorders is still unclear. This study retrospectively documented the clinical phenotype and genotype data of 41 patients with *SCN1A* variants, to explore genotype-phenotypic associations. The results can be used to define the phenotypic spectrum as well as provide a scientific basis for precision-based clinical treatment and genetic counseling for *SCN1A*-related seizure disorders.

## Materials and Methods

### Study Design and Sample Collection

From March 2015 to June 2021, a total of 41 patients with *SCN1A* variants who presented with convulsions at the Department of Neurology, Beijing Children’s Hospital Affiliated to Capital Medical University, China were recruited. The clinical data of these patients and their family members, including sex, age, age of seizure onset, clinical manifestations, seizure evolution, birth history, growth and development history, family history, cranial imaging, electroencephalogram (EEG), and other laboratory examinations, as well as clinical diagnosis, treatment, and follow-up were collected and summarized.

Based on the clinical manifestations, the 41 patients were divided into two groups. In the first, the Dravet syndrome (DS) group, included patients with severe myoclonic epilepsy in infancy (SMEI) and severe myoclonic epilepsy in infancy borderline (SMEB), that also covered intractable childhood epilepsy with generalized tonic-clonic seizures (ICEGTC) (Fujiwara et al., 2003). The second group contained patients of non-Dravet syndrome (non-DS), including genetic epilepsy with febrile seizures plus (GEFS^+^), febrile seizures (FS), febrile seizures plus (FS^+^), and epilepsy not classified as a specific epileptic syndrome. The diagnosis of the patients was made after meeting the specific diagnostic criteria of febrile seizures, epilepsy, and epilepsy syndrome (Commission on Classification and Terminology of the International League Against Epilepsy, 1989).

This study was a retrospective cohort study. The research scheme was approved by the medical ethics committee of Beijing Children’s Hospital Affiliated to Capital Medical University.

### Diagnostic Criteria

Severe myoclonic epilepsy in infancy (SMEI) typically presents with prolonged febrile and afebrile seizures in infants without impaired physical or neurologic development prior to onset. Myoclonic, focal, atypical absence, and atonic seizures present between the ages of 1 and 4 years, This form of epilepsy is usually intractable, and affected children develop epileptic encephalopathy with cognitive, behavioral, and motor impairment. The patient often has a family history of epilepsy or febrile seizures. Severe myoclonic epilepsy in infancy borderline (SMEB) refers to patients who lack several of the key features of SMEI, such as myoclonic seizure or generalized spike-wave discharges in EEG, or exhibit cognitive function impairment to a lesser degree (Scheffer et al., 2009; Guerrini and Oguni, 2011; Wirrell et al., 2017).

Febrile seizures (FS) refer to generalized tonic-clonic seizures (GTCS) with fever that occur between 6 months and 6 years. Febrile seizures that occur outside the normal age limits of classical FS (6 months to 6 years), or afebrile generalized tonic-clonic seizures occur as well as febrile convulsive seizures are referred to as febrile seizures plus (FS^+^). Genetic epilepsy with febrile seizures plus (GEFS^+^) is characterized by a wide phenotypic spectrum, including febrile seizures (FS), FS plus (FS^+^), and FS along with other minor seizure types, although it is considered a familial epilepsy syndrome, but it does not always occur in a familial context, patients with GEFS^+^ phenotypes may have *de novo* variants (Myers et al., 2017; Zhang et al., 2017).

### Statistical Analysis

Data analysis was performed using SPSS version 25.0. Measurement data were expressed as the median while the enumeration data were expressed as the number of cases. For measurement data that presented a normal distribution, a *t*-test was used, else the Mann-Whitney was used for analysis. Enumeration data were analyzed using the chi-square test. Differences were considered statistically significant at *p* < 0.05.

## Results

### Clinical Features of Patients

The clinical information of the 41 patients (26 males and 15 females) with the *SCN1A* variants they harbored is summarized in Tables 1, 2. Of the 41 patients, 21 were classified as DS group (five of SMEI and 16 of SMEB) and 20 patients were non-DS group (four of epilepsy not conforming to definite epilepsy syndrome, 11 of GEFS^+^, four of FS, and one of FS^+^).

**TABLE 1:** Clinical features of patients with SCN1A variant.

Patient No.	Sex	Age at seizure onset	Age at last follow-up	Seizure type	Seizure onset pattern	Seizure evolution	Seizure duration (common/max)	Seizure frequency during 24 h	Birth history	Development history	Family history	EEG (first visit)	Brain MRI	Diagnosis (group)	Medication trials	Seizure condition
1	F	7 m	6 y 6 m	GCS, MS	FS	FS/aFS	1-2 mi n/20 min	1	Normal	Mild retardation	no	Normal	Normal	SMEI (DS)	VPA	seldom
2	M	4 m	5 y	GTCS, AS, FOS	aFS	FS/aFS	20–30 min/30 min	1	Normal	Retardation	no	Abnormal (epileptiform discharges in posterior)	Abnormal (subarachnoid space widening)	SMEI (DS)	VPA LEV CLB KD	Intermittent
3	F	3 m	5 y 4 m	UCS	aFS	FS/aFS	<1 min/20 min	1	Normal	Mild retardation	no	Normal	Abnormal (subarachnoid space widening)	SMEB (DS)	VPA LEV TPM KD	Intermittent
4	F	6 m	4 y	UCS	aFS	aFS (fever after seizure)	2-3 min/30 min	1	Normal	Normal	no	Normal	Normal	SMEB (DS)	LEV VPA	intermittent
5	M	5.7 m	3 y 1 m	GCS	FS	FS/aFS	2–15 min/70 min	1	Normal	Normal	no	Normal	Normal	SMEB (DS)	VPA	intermittent
6	F	6 m	2 y 11 m	sGCS, GCS	FS	FS/aFS	1-2 min/20 min	2	Normal	Normal	no	Normal	Normal	SMEB (DS)	LEV VPA	intermittent
7	M	4 m	3 y 3 m	sGCS	FS/aFS	FS/aFS (seizure during bathing)	10 min/60 min	1	Normal	Mild retardation	Yes/mother	Normal	Abnormal (subarachnoid space widening)	SMEB (DS)	VPA LEV TPM	Intermittent
8	F	4 m	1 y 5 m	sGCS, GCS	aFS (fever after seizure)	FS/aFS	3–5 min/40 min	1	Normal	Retardation	no	Normal	Normal	SMEB (DS)	VPA LEV	Intermittent
9	F	5 m	8 y 1 m	GTCS, FOS	aFS	FS/aFS	1–5 min	1	Normal	Retardation	no	Normal	Normal	SMEB (DS)	VPA	Intermittent
10	M	3 m	1 y 6 m	sGCS, MS	aFS	FS/aFS	3–5 mi n/30 min	1	Normal	Normal	no	Abnormal (generalized epilepstiform discharges)	Abnormal (subarachnoid space widening)	SMEI (DS)	LEV TPM	Intermittent
11	M	12 m	6 y	GTCS, FOS	FS	aFS	3-4min/6 min	1	Normal	Normal	Yes/mother, father	Abnormal (epilepstiform discharges in left posterior region)	Normal	GEFS^+^(non-DS)	LEV	Loss
12	M	10 m	3 y 11 m	GCS	FS	FS/aFS	1-2 min/10 min	2	Normal	Normal	no	Normal	Normal	GEFS^+^(non-DS)	VPA	Loss
13	M	10 m	6 y 6 m	GCS, FOS	FS	aFS	10 min/20 min	1	Normal	Language retardation	yes/father	Abnormal (epilepstiform discharges in left frontal and central region)	Normal	GEFS^+^ (non-DS)	LEV	No seizure
14	F	10 m	4 y	GCS, FOS	FS	aFS	1-2 min/2 min	2	Normal	Normal	Yes/mother, grandmother	Abnormal (generalized epilepstiform discharges)	Normal	GEFS^+^ (non-DS)	NO	Loss
15	F	10 m	6 y 1 m	GCS, FOS	FS	aFS	2-3 min/6 min	1	Normal	Normal	no	Not available	Normal	SMEB (DS)	LEV VPA TPM NZP	No seizure
16	F	5 m	9 y	GCS, FOS	aFS (seizure during bathing)	FS/aFS	1-2 min/2 min	2	Abnormal (post mature delivery postnatal mild hypoxia)	Retardation	no	Normal	Abnormal (poor myelination of periventricular white matter)	SMEB (DS)	LEV VPA TPM NZP	Intermittent
17	M	8 m	7 y 6 m	GTCS	FS	FS/aFS	2-3 min/3 min	5	Abnormal (postnatal mild hypoxia)	Mild retardation	no	Normal	Normal	SMEB (DS)	VPA TPM	Intermittent
18	F	11 m	3 y 5 m	GTCS, aAS	FS/aFS	FS/aFS	4-5 min/5 min	1	Normal	Mild retardation	no	Normal	Abnormal (right hippocampal signal increased slightly)	SMEI (DS)	VPA LEV TPM	intermittent
19	M	10 m	2 y 6 m	GTCS, MS, FOS	FS	FS/aFS	1-2 min/2 min	3	Normal	Mild retardation	no	Abnormal (generalized epilepstiform discharges)	Abnormal (slightly enlarged lateral ventricles)	SMEI (DS)	VPA	seldom
20	M	12 m	4 y 7 m	GTCS	FS	FS/aFS	3-5 min/5 min	1	Normal	Normal	Yes/father, aunt, brother	Abnormal (epileptiform discharges in Rolandic region)	Normal	GEFS^+^ (non-DS)	LEV VPA	No seizure
21	M	7 m	4 y 3 m	GTCS	FS	FS/aFS (seizure during bathing)	3 min/30 min	1	Normal	Mild retardation	no	Normal	Normal	EP (non-DS)	LEV	seldom
22	F	8 m	2 y	GTCS	FS	FS/aFS	5 min/5 min	3	Normal	Normal	no	Boundary (rare spikes infrontal and central regions)	Normal	EP (non-DS)	VPA	intermittent
23	M	9 m	3 y 6 m	GTCS, FOS	FS	aFS	1-2 min/30 min	1	Normal	Normal	Yes/grandfather, sister	Abnormal (small spike in central region)	Normal	GEFS^+^ (non-DS)	VPA LEV	intermittent
24	M	10 m	2 y 10 m	GTCS	FS	aFS(fever after seizure)	1-2 min/5 min	1	Normal	Normal	no	Normal	Normal	EP (non-DS)	No drug	No seizure
25	F	6 m	12 y 11 m	GTCS, FOS	FS	aFS	1-2 min/6 min	3	Normal	Retardation	no	Not available	Normal	SMEB (DS)	VPA LEV	intermittent
26	F	1 y 2 m	5 y	GTCS, FOS	FS	aFS	5-6 min/15 min	1	Normal	Normal	Yes/mother	Abnormal (epilepstiform discharges in posterior region)	Normal	GEFS^+^ (non-DS)	VPA	intermittent
27	M	8 m	7 y 2 m	GTCS	FS	aFS	1-2 min/2 min	1	Normal	Normal	no	Normal	Normal	GEFS^+^ (non-DS)	NO	seldom
28	M	11 m	8 y	GTCS	FS	FS/aFS	2-5 min/5 min	1	Normal	Normal	Yes/father	Normal	Normal	GEFS^+^ (non-DS)	VPA LEV	Loss
29	F	7 m	6 y 4 m	GTCS, FOS	aFS	aFS (seizure usually during infection)	1-2 min/10 min	1	Normal	Normal	no	Normal	Normal	SMEB (DS)	VPA LEV TPM	intermittent
30	F	2 y	4 y 8 m	GTCS, FOS	FS	aFS (fever after seizure)	5-6 min/10 min	1	Normal	Normal	Yes/mother	Abnormal (epileptiform discharges in right frontal, central and temporal regions)	Normal	GEFS^+^ (non-DS)	VPA	Loss
31	M	9 m	2 y 11 m	GCS, aAS	FS	aFS	1-3 min/10 min	1	Normal	Normal	no	Normal	Normal	SMEB (DS)	VPA	intermittent
32	M	1 y 1 m	2 y 7 m	GTCS, FOS	FS	aFS	1-2 min/2 min	1	Normal	Normal	no	Boundary (rare spikes in frontal and central regions)	Abnormal (slightly enlarged lateral ventricles	EP (non-DS)	NO	seldom
33	M	1 y 7 m	8 y	GTCS, FOS	FS	aFS	1 min/1 min	1	Normal	Normal	Yes/father sister	Abnormal (epileptiform discharges in frontal region)	Abnormal (poor myelination of periventricular white matter)	GEFS^+^ (non-DS)	LEV	No seizure
34	M	7 m	10 y 11 m	GTCS, FOS	FS	aFS	1 min/20 min	1	Normal	Mild retardation	Yes/mother	Normal	Normal	SMEB (DS)	VPA LEV	seldom
35	M	10 m	3 y 9 m	GTCS	FS	FS/aFS	1-2 min/30 min	3	Normal	Normal	no	Abnormal (rare epileptiform discharges)	Normal	SMEB (DS)	VPA	seldom
36	M	7 m	4 y 3 m	GTCS, FOS	FS	FS/aFS	2 min/40 min	1	Normal	Mild retardation	Yes/brother	Abnormal (epileptiform discharges in left frontal region)	Normal	SMEB (DS)	VPA LEV TPM NZP	seldom
37	M	10 m	8 y 8 m	GTCS	FS	FS	5 min/5 min	1	Normal	Normal	no	Normal	Normal	FS (non-DS)	NO	No seizure
38	M	2 y	9 y 2 m	GCS	FS	FS	3-5 min/5 min	2	Normal	Normal	no	Not available	Normal	FS^+^ (non-DS)	NO	No seizure
39	M	1 y 5 m	7 y 5 m	GTCS, FOS	FS	FS	2-3 min/3 min	2	Normal	Language retardation	Yes/mother	Not available	Normal	FS (non-DS)	NO	No seizure
40	M	8 m	4 y 8 m	GTCS	FS	FS	1 min/1 min	2	Normal	Normal	no	Normal	Normal	FS (non-DS)	NO	No seizure
41	M	1 y 6 m	5 y	GTCS	FS	FS	1-2 min/2 min	2	Normal	Normal	Yes/brother	Abnormal (epileptiform discharges in posterior)	Normal	FS (non-DS)	NO	No seizure

*AS, absence seizure; aAS, atypical absence seizure; MS, myoclonic seizures; GTCS, generalized tonic–clonic seizure; GCS, generalized clonic seizure; sGCS, secondary generalized clonic seizure; UCS, unilateral clonic seizure; FOS, focal seizure; SMEI, severe myoclonic epilepsy in infancy; SMEB, severe myoclonic epilepsy in infancy borderline; GEFS^+^, genetic epilepsy with febrile seizures plus; FS, febrile seizures; aFS, afebrile seizures; FS^+^, febrile seizures plus; EP, epilepsy; LEV, levetiracetam; TPM, topiramate; VPA, valproate; NZP, nitrazepam; CLB, chlorbazam; KD, ketogenic diet; EEG, electroencephalography; MRI, magnetic resonance imaging.*

**TABLE 2:** Summary of 41 patients’ clinical data.

	DS group	non-DS group
**Sex**		
Male	10	16
Female	11	4
**Age at seizure onset (months)**		
≤6 m	11	0
7–12 m	10	11
≥12 m	0	9
**Mode of onset**		
Onset with febrile seizure	11	20
Onset with afebrile seizue	7	0
Onset with febrile/afebrile seizure	2	0
Onset with fever after seizure	1	0
**Age at first afebrile seizure (months) among onset with febrile seizure**		
≤12 m	2	0
12–36 m	7	6
≥36 m	2	7
Not available	0	2
**Mode of seizure evolution**		
Evolution to afebrile seizure	5	8
Evolution to febrile/afebrile seizure	15	4
Evolution to fever after seizure	1	3
No seizure	0	4
Still febrile seizure	0	1
**Types of seizure**		
Focal	3	0
Focal and secondary generalized	9	9
Generalized	6	11
Focal, generalized and other	3	0
**Duration of seizure**		
<5 min	3	6
≥5 min <10 min	4	8
≥10 min <30 min	6	4
≥30 min	8	2
**Seizure frequency during one day**		
1	15	13
2	2	6
>2	4	1
**Birth history**		
Normal	19	20
Abnormal	2	0
**Family history**		
Yes	3	11
No	18	9
**Developmental delay**		
Yes	13	3
No	8	17
**EEG at initial visit**		
Abnormal	5	9
Normal	14	7
Boundary	0	2
No exam	2	2
**Brain MRI/CT**		
Nonspecific abnormal	7	2
Normal	14	18
**Treatment**		
Single AEDs	6	8
Two AEDs	7	3
Three or more AEDs	8	0
No treatment	0	9
Loss follow-up	0	5
**Seizure outcome**		
Still seizure	20	6
No seizure	1	9
Loss follow-up	0	5

In the DS group, the age of seizure onset ranged from 3 to 11 months, with a median age of 6 months. Of these, 52.4% (11/21) had seizure onset before 6 months of age, and 47.6% (10/21) had seizure onset between 7 months and 1 year of age. In the non-DS group, the age of seizure onset ranged from 7 months to 2 years, with a median age of ten and a half months. Of these, 65% (13/20) had seizure onset between 7 months and 1 year, and 35% (7/20) had seizure onset beyond 1 year of age. The age of seizure onset in the DS group was therefore significantly earlier than that in the non-DS group (*p* < 0.01, Supplementary File 1). Seizure onset patterns differed considerably between the two groups. The patients in the DS group had varied seizure onset patterns since 11 (52.4%) presented with febrile seizure, seven (33.3%) with afebrile seizure and one of them with seizure that occurred during bathing, two (9.5%) with febrile or afebrile seizure, and one (4.8%) with fever after seizure. However, all 20 patients in the non-DS group presented with febrile seizures as their seizure onset pattern. Further, in the DS group, among the 11 patients with febrile seizure onset, two patients evolved to afebrile seizure before the age of 1 year, seven between 1 and 3 years of age, and only two patients after the age of 3 years. In contrast, of the 20 patients in the non-DS group with febrile seizure onset, six patients progressed to afebrile seizure between 1 and 3 years of age, seven after 3 years of age, and none before 1 year of age. It can therefore be inferred that patients in DS group evolve to afebrile seizure earlier than those in non-DS group, however, there was no significant difference in the age of occurrence of the first afebrile seizure between the two groups (*p* > 0.05, Supplementary File 2).

In the DS group, five patients were afebrile at the time of seizure and one developed fever post seizure. The remaining 15 patients later developed febrile or afebrile seizure. Of these, seizures were triggered in four patients by elevated ambient temperature, such as during bathing or vigorous exercise. In the non-DS group, eight patients developed afebrile seizures, one of which occurred during bathing, four presented with febrile or afebrile seizure, three developed fever post seizure, four patients had no recurrence at the time of follow-up, and one patient still experienced febrile seizures. Thus, 58.5% (24/41) of the patients experienced seizures associated with fever or a hot environment with disease progression. This was markedly obvious in the DS group.

The following distribution of type of seizure was seen in the 41 patients included in this study. In the DS group, nine (42.9%) patients presented with generalized or secondary generalized clonic or tonic-clonic seizure, six (28.6%) with generalized clonic or tonic-clonic seizures, three (14.3%) with unilateral clonic seizures, and three (14.3%) with multiple seizures, such as unilateral clonic seizure or generalized tonic-clonic or clonic seizure, myoclonic seizure, and atypical absence seizure. In the non-DS group, nine (45%) patients presented with generalized or secondary generalized tonic-clonic or clonic seizures, and 11 (55%) with generalized tonic-clonic or clonic seizures.

The maximum duration of seizure in the DS group ranged from 2 to 70 min with a median of 20 min, while in the non-DS group it ranged from 1 to 30 min, with a median of 5 min. The duration of seizure was longer than 30 min in eight (38.1%) patients, 10–30 min in six (28.6%) patients, 5–10 min in four (19%) patients, and less than 5 min in three (14.3%) patients in the DS group. In the non-DS group, six (30%) patients had seizure episodes of less than 5 min, eight (40%) patients of 5–10 min, four (20%) patients of 10–30 min, and two (10%) patients of more than 30 min. Seizure duration was therefore significantly longer in the DS group than in the non-DS group (*p* < 0.05, Supplementary File 3). The two groups did not show a significant difference (*p* > 0.05, Supplementary File 4) with respect to frequency of seizure occurrence during a 24 h period, with 15 patients in DS group and 13 patients in non-DS group presenting with a frequency of one seizure, while two patients in DS group and six patients in non-DS group had two seizures, and four patients in DS group and one patient in non-DS group had more than two seizures.

Among the 41 patients, all except for two patients with mild hypoxia at birth had normal antenatal history. Thirteen patients in the DS group and three in the non-DS group had varying degrees of cognitive and motor development retardation. A positive family history of epilepsy or febrile seizures was recorded in 3 and 11 patients in the DS group and non-DS group, respectively.

Brain MRI of nine patients (seven in the DS group and two in the non-DS group) showed non-specific abnormalities, including four patients in DS group showed subarachnoid space widening, one patient in DS group and one patient in non-DS group showed poor myelination of periventricular white matter, one patient in DS group showed marginally higher hippocampal signal, and one in DS group and one in non-DS group showed slightly enlarged lateral ventricles. At least one electroencephalography (EEG) examination was performed on 37 patients. At the first visit, but not necessarily early in the course of the disease, EEG abnormalities including focal and generalized epileptiform discharges were seen in 14 patients (five in the DS group and nine in the non-DS group). Another two patients in the non-DS group had borderline abnormal changes and the remaining 21 patients (14 in the DS group and seven in the non-DS group) had normal EEG. In the DS group, the EEG of 14 patients were normal, of which eight patients were examined EEG before the age of 1 year at the initial stage of seizure onset, six patients were examined between 1 and 2 years old. The EEG results of remaining two patients in the DS group were unknown, which were not provided by their parents at the first visit, and the other two patients in non-DS group were diagnosed with FS and FS^+^, respectively without examination of EEG.

All patients were followed up with outpatient services or telephonic consultations. In the DS group, the age at last follow-up ranged from 1 year and 5 months to 12 years and 11 months, with a median of 4 years and 3 months. In the non-DS group, it ranged from 2 to 10 years and 11 months, with a median of 5 years. There was no significant difference in age at last follow-up between the two groups (*p* > 0.05, Supplementary File 5). At the time of follow up, 26 patients, including 20 (20/21, 95.2%) patients in the DS group and six (6/20, 30%) patients in the non-DS group continued to experience seizures. Another 10 patients, nine (9/20, 45%) patients in the non-DS group and one (1/21, 4.8%) patient in the DS group no longer suffer from seizures at present, and the remaining five were lost to follow-up. In the DS group, eight (8/21, 38.1%) patients were treated with more than three antiepileptic drugs, seven (7/21, 33.3%) with two antiepileptic drugs, and six (6/21, 28.6%) with only one antiepileptic drug. By the time of follow-up, only one patient in the DS group had not experienced seizures for 8 months after treatment with four antiepileptic drugs. In the non-DS group, eight (8/20, 40%) patients were not prescribed drugs and advised to prevent hyperthermia alongside temporary administration of sedatives to prevent convulsions during fever, six of whom experienced no seizures, and two experienced seldom seizures. The remaining two patients, one of whom no longer has seizures, and the other who rarely experience seizures, were prescribed two antiepileptic drugs. Another five patients had one antiepileptic drug, two of whom experienced no seizures, and three experienced intermittent seizures.

### Genetic Characteristic of *SCN1A* Variants Identified in This Study

Details of the identified *SCN1A* variants are summarized in Tables 3, 4. Among the 41 patients, 31(75.6%) had *de novo* variants, including 20 patients in the DS group and 11 in the non-DS group. Inherited variants were seen in nine (22%) patients, including one patient in the DS group and eight in the non-DS group, all of whom reported a family history of febrile seizure or epilepsy. Among the patients with *de novo* variants, four (two in the DS group and two in the non-DS group) had a positive family history of febrile seizures despite not having inherited a pathogenic variant. Additionally, the origin of the variant identified in a single patient could not be verified due to unavailability of the father’s blood sample.

**TABLE 3:** Genetic characteristic of 41 *SCN1A* variants identified in this study.

No./Phenotype	Exon	cDNA*	Variant	Location in	Mutation type	Transmission	Reported mutation
				protein			(references)
							(previous diagnosis)
1/SMEI	4	c.563A > C	p.D188A	DIS2-DIS3	Missense	De novo	No
2/SMEI	26	c.4571delC	p.P1524Lfs*15	DIIIS6-DIVS1	Frameshift	De novo	No
3/SMEB	26	c.5035delC	p.L1679X	DIVS5	Nonsense	De novo	No
4/SMEB	16	c.3111dupC	p.F1038Lfs*3	DIIS6-DIIIS1	Frameshift	De novo	No
5/SMEB	26	c.4439G > T	p.G1480V	DIIIS6-DIVS1	Missense	De novo	Yes (Harkin et al., 2007) MAE
6/SMEB	4	c.272T > C	p.I91T	H3N^+^-DI	Missense	De novo	Yes (Sun et al., 2008) SMEI
7/SMEB	13	c.1837C > T	p.R613X	DIS6-DIIS1	Nonsense	Maternal	Yes (Kearney et al., 2006) SMEI
8/SMEB	25	c.4301G > A	p.W1434X	DIIIS5-DIIIS6	Nonsense	De novo	Yes (Zucca et al., 2008) SMEI
9/SMEB	19	c.3759dupA	p.Y1254Ifs*3	DIIIS2	Frameshift	De novo	No
10/SMEI	23	c.3981delA	p.L1327Ffs*7	DIIIS4	Frameshift	De novo	No
11/GEFS^+^	1	c.1852C > T	p.R618C	DIS6-DIIS1	Missense	Maternal	Yes (Brunklaus et al., 2015) GEFS^+^ SMEI
12/GEFS^+^	15	c.2732_2733delinsAA	p.L911Q	DIIS5-DIIS6	Missense	De novo	No
13/GEFS^+^	21	c.4112G > C	p.G1371A	DIIIS5-DIIIS6	Missense	De novo	No
14/GEFS^+^	2	c.364A > G	p.I122V	H3N^+^-DI	Missense	Maternal	Yes (Till et al., 2020) SMBI
15/SMEB	18	c.2791C > T	p.R931C	DIIS5-DIIS6	Missense	De novo	Yes (Ohmori et al., 2002) SMEI
16/SMEB	28	c.4762T > C	p.C1588R	DIVS2	Missense	De novo	Yes (Marini et al., 2007) SMEI
17/SMEB	18	c.2946+2T > C	(splicing)	DIIS6	Splice site	De novo	No
18/SMEI	12	c.2134C > T	p.R712X	DIS6-DIIS1	Nonsense	De novo	Yes (Sugawara et al., 2002) SMEI
19/SMEI	15	c.2134C > T	p.R712X	DIS6-DIIS1	Nonsense	De novo	Yes (Sugawara et al., 2002) SMEI
20/GEFS^+^	28	c.4741A > G	p.I1581V	DIVS2	Missense	Paternal	No
21/EP	6	c.706A > T	p.I236F	DIS4-DIS5	Missense	De novo	No
22/EP	20-29del				Partial exon deletion	De novo	No
23/GEFS^+^	28	c.5218G > T	p.D1740Y	DIVS5-DIVS6	Missense	Paternal	No
24/EP	2q24.3del				2q24.3 deletion	De novo	No
25/SMEB	9	c.825T > G	p.N275K	DIS5-DIS6	Missense	De novo	No
26/GEFS^+^	7	c.493T > C	p.Y165H	DIS2	Missense	Maternal	Yes (Fernández-Marmiesse et al., 2019) SMBI
27/GEFS^+^	19	c.3867_3869delCTT	p.1289delF	DIIIS3	Deletion	De novo	Yes (Ohmori et al., 2006) SMBI
28/GEFS^+^	14	c.2576G > A	p.R859H	DIIS4	Missense	De novo	Yes (Volkers et al., 2011) GEFS^+^
29/SMEB	5	c.680T > G	p.I227S	DIS4	Missense	De novo	Yes (Nabbout et al., 2003) SMEI
30/GEFS^+^	26	c.5770C > G	p.R1924G	DIVS6-CO2-	Missense	Maternal	No
31/SMEB	24	c.4049T > C	p.V1350A	DIIIS4	Missense	De novo	No
32/EP	18	c.2735T > C	p.F912S	DIIS5-DIIS6	Missense	De novo	No
33/GEFS^+^	6	c.G709C	p.V237L	DIS4-DIS5	Missense	Paternal	No
34/SMEB	21	c.4168G > A	p.V1390M	DIIIS5-DIIIS6	Missense	De novo	Yes (Rilstone et al., 2012) SMEI
35/SMEB	17	c.2585G > T	p.R862L	DIIS4	Missense	De novo	No
36/SMEB	28	c.5108A > C	p.D1703A	DIVS5-DIVS6	Missense	De novo	No
37/FS	18	c.3643G > T	p.V1215F	DIIS6-DIIIS1	Missense	De novo	No
38/FS^+^	20	c.3973A > G	p.R1325G	DIIIS4	Missense	De novo	No
39/FS	29	c.5389G > T	p.A1797S	DIV-CO2	Missense	Unknown	No
40/FS	26	c.5078C > A	p.A1693D	DIVS5-DIVS6	Missense	De novo	No
41/FS	28	c.4787G > A	p.R1596H	DIVS2-DIVS3	Missense	Paternal	Yes (Zuberi et al., 2011) GEFS^+^

DIS1, domain 1 segment 1; SMEI, severe myoclonic epilepsy in infancy; SMEB, severe myoclonic epilepsy in infancy borderline; GEFS^+^, genetic epilepsy with febrile seizures plus; EP, epilepsy not classified as a specific epileptic syndrome; FS^+^, febrile seizures plus; FS, febrile seizures. *Coding DNA reference sequence NM_001165963 does not contain intron sequences.

**TABLE 4:** Summary of 41 patients’ genetic data.

ITEM\case	DS group	Non-DS group	Total
	21	20	41
Genetic variation	1	8	9
De novo	20	11	31
Unknown	0	1	1
Missense mutation	11	17	28
Nonsense mutation	5	0	5
Frameshift	4	0	4
Splice site	1	0	1
Deletion mutation	0	1	1
Partial deletion of SCN1A exon	0	1	1
2q^24.3^ deletion	0	1	1

The distribution of variant type in the DS group included nonsense variants in five patients, frameshift variants in four, a splice site variant in one, and missense variants in the remaining eleven. Missense variants were found in 17 patients, and the remaining three had a deletion, a partial deletion of *SCN1A* exon 20–29, and a 2q^24.3^ deletion each in the non-DS group.

**FIGURE 1:**
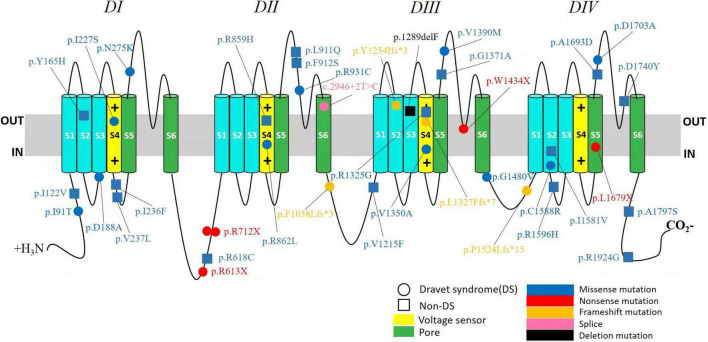
Structure of the human Nav1.1 channel and the location of *SCN1A* variants identified in this study. Nav1.1 channels α subunit consist of four homologous domains (DI–DIV), each with six transmembrane segments (S1–S6). The fourth segment (S4) of each domain functions as a voltage sensor. The S5 and S6 segments of each domain make up the pore of the channel, and the connecting loop between S5 and S6 is the pore loop. The identified variants in DS group were depicted with circle and in non-DS group with square. Missense variants are shown in blue. Nonsense variants are shown in red. Frameshift variants are shown in orange. Splice site variant is shown in pink and deletion variant in black.

**FIGURE 2:**
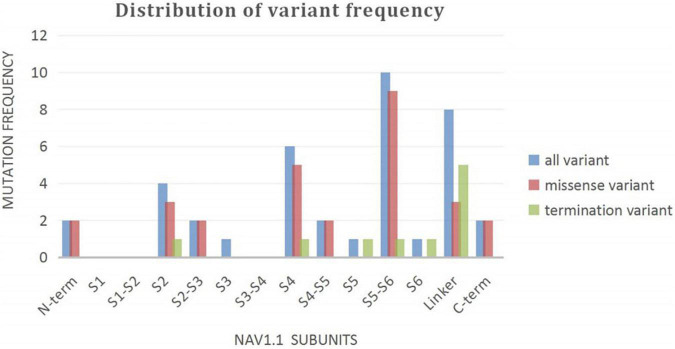
Distribution of variant frequency of 39 patients with point variant in our study.

**FIGURE 3:**
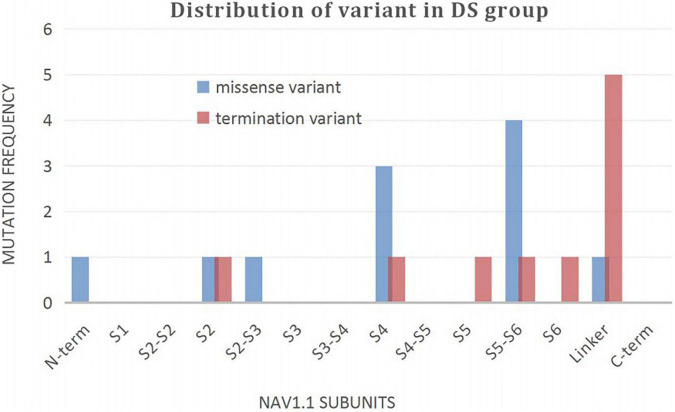
Distribution of variant in DS group.

**FIGURE 4:**
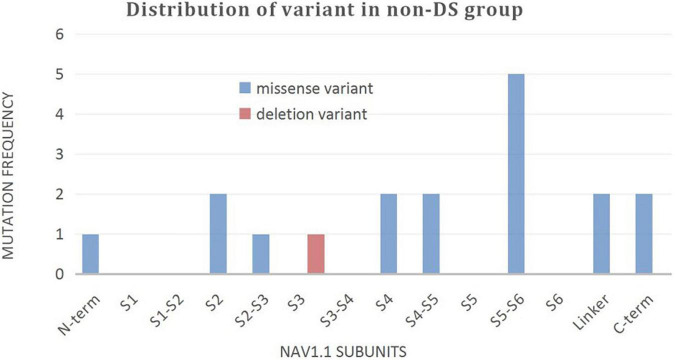
Distribution of variant in non-DS group.

A wide distribution of the 39 *SCN1A* point variants identified across all domains of the sodium channels were observed (Figure 1). Of these, 10 mapped to the sodium channel pore-forming region (S5–S6), six to the voltage sensor region (S4), eight to the linker regions, and the remaining 15 were scattered evenly throughout the sodium channels Nav1.1 (Figure 2). Among the eleven missense variants in the DS group, three were located in the S4 region, four in the S5–S6 region, and the rest were evenly distributed. Of the 10 termination variants, including nonsense, frameshift, and splice site variants, five were located in the linker region, and the rest were distributed evenly (Figure 3). In the non-DS group, 17 patients harbored missense variants, of which five were located in the S5–S6 region, two in the S4 region, and the remaining were evenly distributed (Figure 4).

## Discussion

The *SCN1A* gene was first associated with genetic (formerly generalized) epilepsy with febrile seizures plus (GEFS+) (Escayg et al., 2000). Subsequently, it was discovered that the vast majority of patients with Dravet syndrome harbored *SCN1A* variants with an increasing number of *SCN1A* variants being reported. To date, the clinical spectrum of epilepsies related to *SCN1A* variants encompass various phenotypes, the most common being SMEI and SMEB, and a small proportion being GEFS^+^, including FS and FS^+^. Other phenotypes included epilepsies not classified as a definite epileptic syndrome and rare early onset developmental and epileptic encephalopathy (Sadleir et al., 2017).

Although the 41 patients in our study presented with the same complaint of intermittent convulsions with or without fever in the early stage of the disease, other clinical manifestations were not similar. Age of seizure onset in the DS group was significantly earlier than that of the non-DS group, since all the patients were below 1 year of age, and most were symptomatic before 6 months of age. The pattern of onset in the DS group was either febrile or afebrile seizures, or an alternate occurrence of the two. In the non-DS group the only pattern of onset was febrile seizures. Patients in the DS group initially showed unilateral clonic seizure or secondary generalized clonic or tonic-clonic seizures, or generalized clonic or tonic-clonic seizures. However, those in the non-DS group showed generalized or secondary generalized clonic or tonic-clonic seizures without unilateral clonic seizures. Longer duration of seizures as well as a greater tendency to be prone to status epilepticus was reported from patients in the DS group than those in the non-DS group. The vast majority of patients in the DS group experienced seizures with a frequency of one every 24 h, however, some in the non-DS group experienced more than two seizures in the same time period and demonstrated some clustered features. While there was no significant difference between the two groups, the age of first afebrile seizure in patients with febrile seizure at onset was slightly earlier in the DS group than in the non-DS group. To summarize, the clinical manifestations of both groups are specific and vary in terms of severity and complexity.

In spite of phenotypic differences, several core features remained consistent. Seizure-related fever was observed in 34 patients (83%) at onset, and the remaining seven patients with afebrile onset of seizure eventually developed febrile seizures in the course of the disease. In addition, 32 patients (78%) experienced seizure durations of more than 5 min, and 20 patients (50%) had prolonged seizures that lasted longer than 10 min, especially in the DS group. Consequently, in concordance with previous findings, fever-related and/or prolonged seizures are prominent phenotypes associated with *SCN1A* variants (Guerrini and Oguni, 2011).

The phenotype of *SCN1A* variant is a continuous disease spectrum ranging from the mild self-limited and drug-reactive diseases, such as GEFS^+^, FS, and FS^+^ to the severe drug-refractory developmental epileptic encephalopathies (DEE), including DS and other rare phenotypes such as myoclonic-atonic epilepsy (MAE), epilepsy of infancy with migrating focal seizures (EIMFS), and early onset *SCN1A*- related DEE, which have been reported in rare cases (Wallace et al., 2001; Carranza Rojo et al., 2011; Freilich et al., 2011). With the wide application of gene sequencing technology, some focal epilepsies have been found to be associated with *SCN1A* variants (McDonald et al., 2017; Bisulli et al., 2019). Thus, the phenotypes of *SCN1A* variants possess significant heterogeneity. Many of the differences between DS and non-DS are a function of the definitions of the disease. They have common genetic etiology and are a continuous entity of a disease with a wide range of seizure types and severities. Sometimes, it is very hard to separate adjacent phenotypes with a line.

As many as 2,127 diverse variations in the *SCN1A* have been reported that map to almost every domain of the voltage-gated sodium channel alpha 1 subunit protein, resulting in a large assortment of potential alterations in channel function. Phenotypic variations as the consequence of *SCN1A* variations depend on a variety of factors including position, type, and the category of functional change induced by variants, as well as the role of other modifier genes and environmental effects (Meng et al., 2015; Fang et al., 2019). The main etiology of *SCN1A* associated epileptic phenotypes is closely related to its functional changes. The functional researches of *SCN1A* variants have confirmed that loss-of-function leading to haploinsufficiency is the main effect of both Dravet syndrome and GEFS^+^, although for some variants, mixed loss- and gain-of-function effects and for few variants a net gain-of-function have been observed (Mantegazza and Broccoli, 2019). Recent study has investigated that *SCN1A* T226M variant presents gain-of-function in early infantile development and epileptic encephalopathy which is far more severe than typical Dravet syndrome (Berecki et al., 2019). Previous studies have demonstrated the correlation of clinical severity with variant type, with missense changes resulting in milder and truncations causing severe epilepsy (Kanai et al., 2004; Stafstrom, 2009). So, genotypes determine phenotypes, the relationship appears unexpected complexity involving numerous known and unknown factors related to intrinsically complex pathophysiologic responses as well as environmental factors, which yet remain to be fully disentangled.

In our study, 10 patients in the DS group carried protein termination variants, including five nonsense, four frameshift, and one splice site variant. Termination of protein translation resulting in a short truncated protein is predicted to cause complete loss of sodium channel function and a consequent severe phenotype. The remaining eleven patients harbored missense variants, of which seven were located within functionally important domains including the sodium channel ion pore and voltage sensor regions and still had seizures up to the last follow-up, only one patient treated with four kinds of antiepileptic drugs without seizures for 8 months. Our results are consistent with those of previous studies (Ceulemans et al., 2004; Kanai et al., 2004; Zuberi et al., 2011) and demonstrate a correlation between genotype (such as the nature and location of variants) and phenotype. Of the variants associated with severe phenotypes, truncation variants are often evenly distributed in the sodium channels, while missense variants are mostly concentrated around voltage sensors and channel pores (Zuberi et al., 2011). Our results demonstrate a higher distribution of termination variants in the linker region. Given that this is a novel finding, it will be interesting to validate it in a large sample size study. About truncation variants, another situation requires attention. It is identified that few DS patients are caused by poison exons that are naturally occurring and highly conserved exons, these poison exons contain a premature termination codon, alternative splicing of such an exon is predicted to lead to nonsense mediated decay, decreasing the amount of protein produced (Carvill and Mefford, 2020; Aziz et al., 2021). Therefore, the results of nonsense mediated decay are similar to those of truncation variants.

Among the 17 patients with missense variants in the non-DS group, seven carried variants that were located within the sodium channel ion pore and voltage sensor regions. Except two patients lost to follow-up, only two patients with the phenotypes of GEFS^+^ and epilepsy not classified as a specific epileptic syndrome continued to experience intermittent seizures, the other three patients, including one case of GEFS^+^, FS and FS^+^, respectively, fared better with stoppage of seizure episodes. We were therefore able to conclude that variant location in a functionally important domain of the sodium channel need not necessarily translate into a serious phenotype. The reason why certain missense variations are linked to severe phenotypes, while others are not, is not well elucidated. Similarly, in the DS group, two patients with identical genotypes carrying the same nonsense variants had significantly different phenotypes, one with mild epilepsy and the other suffering from severe disease. This strongly suggests the influence of other factors including modifier genes, environmental effects, and brain development in determining the phenotypic expression of the disease (Stafstrom, 2009; Parihar and Ganesh, 2013). Genetic modifiers are genes distinct from the primary mutation that modulate the severity of the disease phenotype (Kearney, 2011). The studies identified several genetic modifiers such as *Hlf*, the gene encoding hepatic leukemia factor, *Cacna1g*, the gene encoding Cav3.1 and *Gabra2*, the gene encoding the GABA_*A*_ receptor α2 subunit that influence the phenotypes and severity of *Scn1a*^+/−^ mouse model of Dravet syndrome (Hawkins and Kearney, 2016; Calhoun et al., 2017; Hawkins et al., 2021). In addition, it is also reported that variants in *Scn2a, Kcnq2*, and *Scn8a* can dramatically influence the phenotype of mice carrying the *Scn1a*-R1648H mutation (Hawkins et al., 2011). The ability to study the complex genetic interactions in model organisms contributes to our understanding of the genetic factors that influence neurological disease and suggest candidate genes for follow-up study in human patients.

With the researches of the pathogenic mechanism of *SCN1A*-related epilepsy, especially the functional changes and genetic modifiers, there are some novel therapeutic approaches that target seizure control through genetic modulation have emerged. Due to *SCN1A* haploinsufficiency of most DS patients, the first precision therapy was antisense oligonucleotides (ASO) which can restore functional *SCN1A* mRNA and NaV1.1 levels. It was developed using Targeted Augmentation of Nuclear Gene Output (TANGO) technology (Lim et al., 2020). Another gene therapy focuses on adeno-associated virus (AAV)-delivered gene modulation. For example, an adeno-associated virus serotype 9 (AAV9) vector-based, GABAergic neuron-selective, which can upregulate endogenous *SCN1A* gene expression to prevent GABAergic neurons disinhibition (Isom and Knupp, 2021). The treatments of genetic epilepsy will no longer be solely symptomatic to control seizures, but will be transformed into genomics-driven personalized therapy for underlying molecular defects or its consequences.

Our findings should be interpreted in the context of certain limitations. This retrospective study only includes patients who came to our hospital for diagnosis and treatment, which may result in some bias. Additionally, our sample size is small and follow-up duration was short. These limitations can be overcome by further long term follow up studies in large cohorts. Our findings will help to further understand the clinical characteristic significance of *SCN1A* variants. The phenotypes of *SCN1A* associated seizure disorder are significantly heterogeneous over a continuous spectrum from mild to severe with both commonness and individuality. The effect of variants on phenotype is not completely determined by location and type, but also due to functional changes, genetic modifiers as well as other known and unknown factors, therefore genotype-phenotype predictions cannot be easily made. Evaluation of patients in the early stage of disease, with respect to clinical manifestations and *SCN1A* variant features is crucial to assess disease progression for early identification of patients who may benefit most from precise medical intervention.

## Data Availability Statement

The original contributions presented in the study are included in the article/Supplementary Material, further inquiries can be directed to the corresponding author/s.

## Ethics Statement

The studies involving human participants were reviewed and approved by the medical ethics committee of Beijing Children’s Hospital Affiliated to Capital Medical University. Written informed consent to participate in this study was provided by the participants’ legal guardian/next of kin. Written informed consent was obtained from the minor(s)’ legal guardian/next of kin for the publication of any potentially identifiable images or data included in this article.

## Author Contributions

CC conceptualized and designed the study, drafted the initial manuscript, and reviewed and revised the manuscript. CC, FF, XW, JL, XHW, and HJ collected data and carried out the initial analyses. All authors approved the final manuscript as submitted and agreed to be accountable for all aspects of the work.

## Conflict of Interest

The authors declare that the research was conducted in the absence of any commercial or financial relationships that could be construed as a potential conflict of interest.

## Publisher’s Note

All claims expressed in this article are solely those of the authors and do not necessarily represent those of their affiliated organizations, or those of the publisher, the editors and the reviewers. Any product that may be evaluated in this article, or claim that may be made by its manufacturer, is not guaranteed or endorsed by the publisher.
