# Supplementary material for: Phenotypic and Genotypic Characteristics of SCN1A Associated Seizure Diseases
Source: Front Mol Neurosci. 2022 Apr 28;15:821012. doi: 10.3389/fnmol.2022.821012 (PMC9096348; doi:10.3389/fnmol.2022.821012)
Supplement: Supplementary file 1 [file Table_1.docx]

Supplementary File 1 Age at seizure onset/month

| DS group | non-DS group |
| --- | --- |
| 7 | 12 |
| 4 | 10 |
| 3 | 10 |
| 6 | 10 |
| 5.7 | 12 |
| 6 | 7 |
| 4 | 8 |
| 4 | 9 |
| 5 | 10 |
| 3 | 14 |
| 10 | 8 |
| 5 | 11 |
| 8 | 24 |
| 11 | 13 |
| 10 | 19 |
| 6 | 10 |
| 7  9  7  10 | 24 |
| 7 | 17 |
|  | 8 |
|  | 18 |
|  |  |
|  |  |
|  |  |
| *P<0.01* | |

*p* Value derived using Mann-Whitney U test.

Significant, *p<0.05*
