# Supplementary material for: Phenotypic and Genotypic Characteristics of SCN1A Associated Seizure Diseases
Source: Front Mol Neurosci. 2022 Apr 28;15:821012. doi: 10.3389/fnmol.2022.821012 (PMC9096348; doi:10.3389/fnmol.2022.821012)
Supplement: Supplementary file 2 [file Table_2.docx]

Supplementary File 2 the age of evolving to afebrile seizure

| DS group | Non-DS group |
| --- | --- |
| 25 | 72 |
| 12 | 47 |
| 11 | 30 |
| 45 | 40 |
| 30 | 18 |
| 14 | 39 |
| 36 | 28 |
| 29  27  30 | 60 |
| 30 | 15 |
| - | 54 |
| - | 25 |
| - | 24 |
| - |  |
| - |  |
| *p=0.077* | |

“-“, The child had no afebrile convulsion

*p* Value derived using Independent-Samples T Test.

Significant, *p<0.05*
