# Supplementary material for: Phenotypic and Genotypic Characteristics of SCN1A Associated Seizure Diseases
Source: Front Mol Neurosci. 2022 Apr 28;15:821012. doi: 10.3389/fnmol.2022.821012 (PMC9096348; doi:10.3389/fnmol.2022.821012)
Supplement: Supplementary file 3 [file Table_3.docx]

Supplementary File 3 The duration of seizure/minute

| DS group | Non-DS group |
| --- | --- |
| 20 | 6 |
| 30 | 10 |
| 20 | 20 |
| 30 | 2 |
| 70 | 5 |
| 20 | 30 |
| 60 | 5 |
| 40 | 30 |
| 5 | 5 |
| 30 | 15 |
| 6 | 2 |
| 2 | 5 |
| 3 | 10 |
| 5 | 2 |
| 2 | 1 |
| 6 | 5 |
| 10 | 5 |
| 10  20  30 | 3  1  1 |
| 40 |  |
|  |  |
|  |  |
|  |  |
| *P<0.01* | |

*p* Value derived using Mann-Whitney U test.

Significant, *p<0.05*
