# Supplementary material for: Phenotypic and Genotypic Characteristics of SCN1A Associated Seizure Diseases
Source: Front Mol Neurosci. 2022 Apr 28;15:821012. doi: 10.3389/fnmol.2022.821012 (PMC9096348; doi:10.3389/fnmol.2022.821012)
Supplement: Supplementary file 4 [file Table_4.docx]

Supplementary File 4 the seizure frequency during 24 hours/number

| DS group | non-DS group |
| --- | --- |
| 1 | 1 |
| 1 | 2 |
| 1 | 1 |
| 1 | 2 |
| 1 | 1 |
| 2 | 1 |
| 1 | 3 |
| 1 | 1 |
| 1 | 1 |
| 1 | 1 |
| 1 | 1 |
| 2 | 1 |
| 5 | 1 |
| 3 | 1 |
| 1 | 1 |
| 3 |  |
| 1 |  |
| 1  1  3 | 1 |
| 1 | 2 |
|  | 2 |
|  | 2 |
|  | 2 |
| *p=0.95* | |

*p* Value derived using Mann-Whitney U test.

Significant, *p<0.05*
