# Supplementary material for: Phenotypic and Genotypic Characteristics of SCN1A Associated Seizure Diseases
Source: Front Mol Neurosci. 2022 Apr 28;15:821012. doi: 10.3389/fnmol.2022.821012 (PMC9096348; doi:10.3389/fnmol.2022.821012)
Supplement: Supplementary file 5 [file Table_5.docx]

Supplementary File 5 Age at last follow-up/year

| DS Group | non-DS Group |
| --- | --- |
| 6.50 | 6.50 |
| 5.00 | 4.58 |
| 5.33 | 5.75 |
| 4.00 | 2.00 |
| 3.08 | 3.50 |
| 2.92 | 2.83 |
| 3.25 | 5.00 |
| 1.42 | 7.17 |
| 7.75 | 2.58 |
| 1.50 | 8.00 |
| 6.08 | 8.67 |
| 9.00 | 9.17 |
| 7.50 | 7.42 |
| 3.42 | 4.67 |
| 2.50 | 5.00 |
| 12.92 |  |
| 6.33 |  |
| 2.92  10.92  3.75 |  |
| 4.25 |  |
| *p=0.774* | |

*p* Value derived using Independent-Samples T Test.

Significant, *p<0.05*
